# Perturbed Glucose Metabolism: Insights into Multiple Sclerosis Pathogenesis

**DOI:** 10.3389/fneur.2014.00250

**Published:** 2014-12-01

**Authors:** Deepali Mathur, Gerardo López-Rodas, Bonaventura Casanova, Maria Burgal Marti

**Affiliations:** ^1^Department of Functional Biology, University of Valencia, Valencia, Spain; ^2^Department of Biochemistry and Molecular Biology, INCLIVA Biomedical Research Institute, University of Valencia, Valencia, Spain; ^3^Hospital Universitari i Politècnic La Fe, València, Spain; ^4^Multiple Sclerosis Laboratory, Department of Biomedicine, Prince Felipe Research Center, Valencia, Spain

**Keywords:** brain glucose metabolism, cell-specific mechanisms, mitochondrial defects, multiple sclerosis, neurodegenerative diseases

## Abstract

Multiple sclerosis (MS) is a complex debilitating disease of the central nervous system (CNS) perceived to result from the autoimmune effect of T cells in damaging myelin sheath. However, the exact pathogenesis of the disease remains elusive. Initial studies describing the possibility of defective pyruvate metabolism in MS were performed in 1950s. The group observed elevated blood pyruvate level in both fasting and postprandial times in MS patients with relapse. Similarly, other investigators also reported increased fasting pyruvate level in this disease. These reports hint to a possible abnormality of pyruvate metabolism in MS patients. In addition, increase in levels of Krebs cycle acids like alpha-ketoglutarate in fasting and citrate after glucose intake in MS patients further strengthened the connection of disturbed pyruvate metabolism with MS progression. These studies led the investigators to explore the role of disturbed glucose metabolism in pathophysiological brain function. Under normal circumstances, complex molecules are metabolized into simpler molecules through their respective pathways. Differential expression of genes encoding enzymes of the glucose metabolic pathway in CNS may result in neurological deficits. In this review article, we discuss the studies related to disturbed carbohydrate metabolism in MS and other neurodegenerative diseases. These observations open new perspectives for the understanding of metabolic dynamics in MS yet many puzzling aspects and critical questions need to be addressed. Much more research is required to fully unravel the disease mechanism, and a proper understanding of the disease could eventually lead to new treatments.

## Introduction

Multiple sclerosis (MS) is a chronic inflammatory disease of probably immune origin affecting more than two million people worldwide. Demyelinated plaque, inflammatory infiltrates, accumulation of antibodies, and complement proteins are the pathological hallmarks of MS (1). It is believed that immune cells particularly T cells penetrate the brain and mistakenly recognize myelin tissue as foreign and damage it. The inflammatory cells including microglia, macrophages; antibodies, cytokines, complement system, and others enhance the damaging effect. The process of demyelination is associated with axonal degeneration that underlies neurological deficits in MS (2). The lesions formed as a consequence of neuroaxonal injury are found in both white and gray matter of the central nervous system (CNS) (3). Although research underscores the role of immunological, genetic, and environmental pathologic influences, the exact mechanisms underlying MS pathology are yet uncertain.

The disease course often begins with clinically isolated syndrome involving optic nerve, brain stem or spinal cord. The 70–80% of these patients experience relapsing-remitting events, which at later stage are transformed into a secondary progressive stage that causes irreversible neurologic worsening. This suggests that the pathophysiology of progression is not solely inflammatory in nature (4). To prevent disease progression investigators have explored a possible role of energy metabolism in the CNS. Metabolic disturbances have been implicated in neurodegenerative disorders including Alzheimer’s, Huntington’s, and Parkinson’s diseases. In demyelinating diseases particularly MS, investigations on the contribution of disturbed glucose metabolism in MS pathology are limited. However, the existing literature hints to a connection between disturbed glucose metabolism and MS pathogenesis. In this context, this article will first focus on some basic aspects of brain’s energy balance, its regulation at cellular level, and its role in normal and diseased conditions with a special focus on MS. Before we discuss the cell-specific mechanisms underlying brain energy metabolism, it seems pertinent to briefly review the metabolic pathways, which include glycolysis, the tricarboxylic acid (TCA) cycle, and the pentose phosphate pathway (PPP). These metabolic pathways are similar in brain and other tissues.

### Glycolysis

Glycolysis (Embden–Meyerhof pathway) is the metabolism of glucose to pyruvate. Four ATP molecules are produced in the processing of glucose to pyruvate, where two ATP molecules are consumed in the pathway resulting in a net production of two ATP molecules per glucose molecule. In the absence of oxygen (anaerobic conditions), pyruvate is converted into lactate, allowing the regeneration of nicotinamide adenine dinucleotide (NAD^+^), which is necessary to maintain a continued glycolytic flux. Without the regeneration of NAD^+^ the pathway could not have continued beyond glyceraldehyde 3-phosphate.

### Tricarboxylic acid cycle

Under aerobic conditions, pyruvate undergoes oxidative decarboxylation to yield acetyl-CoA in a reaction catalyzed by pyruvate dehydrogenase. Acetyl-CoA in the presence of citrate synthase condenses with oxaloacetate and forms citrate. This is the first step of the TCA cycle in which three molecules of NADH are formed from NAD + and one molecule of FADH2 is formed from flavin adenine dinucleotide (FAD) through four oxidation–reduction steps. The reducing equivalents NADH and FADH2 transfer their electrons to molecular oxygen via mitochondrial electron transport chain (ETC). There are five enzyme complexes, denoted as I–V that forms the ETC in mitochondria. Electron transfer from complexes I and II to complex III and from complex III to complex IV is accomplished by co-enzymes ubiquinone and cytochrome c. During this process, protons are transported across the inner mitochondrial membrane to the intermembrane space to generate an electrochemical gradient. The enzyme ATP synthase utilizes this energy and produces ATP.

### Pentose phosphate pathway

Overall, glucose is completely oxidized to carbon dioxide and water via three synchronized pathways namely glycolysis, the TCA cycle, and the ETC, and finally produces energy in the form of ATP. However, there are conditions when extra metabolic energy is required in the form of reducing power in addition to ATP. These are the situations when precursors are in a more oxidized state than the products. In PPP, glucose 6-phosphate is converted into ribulose 5-phosphate utilizing two molecules of NAD+. Thus, when ample amount of energy is required, the level of NADPH falls down and the pathway is activated to generate more reducing equivalents.

## Glucose Serving as the Main Fuel in Brain

The human brain represents only 2% of the body weight, yet 25% of total body glucose is utilized by it. Energy requirement is highest in neurons of adult brain (5). Therefore, a continuous supply of glucose is needed from bloodstream into the brain. With a few exceptions, glucose is the main source of energy in mammalian brain (6). Nevertheless, there are certain circumstances when brain uses ketone bodies as energy source including starvation, during strenuous exercise and development (7). Metabolism of glucose yields energy in the form of ATP that is used for neuronal and non-neuronal cell survival and generation of neurotransmitters. Therefore, tight regulation of glucose metabolism is necessary for normal brain physiology and perturbation in any step of its regulation pathway may form the pathophysiological nexus for many brain disorders.

## Cell-Specific Mechanisms Underpinning Brain Energy Metabolism

### Glia and vascular endothelial cells – role in brain energy metabolism

Neurons are usually regarded the most important cells of the CNS taking part in energy metabolism. However, other cells like glial and vascular endothelial cells also play a critical role in the distribution of energy substrates to neurons. Glial cells constitute nearly half of the brain volume. Out of many different cell types in the brain, neurons represent only a small proportion for glucose utilization. It has been seen that the presence of specialized end-feet processes makes astrocytes the first cellular barrier that glucose entering the brain parenchyma come across and makes them a probable site of glucose uptake and energy substrate distribution. Besides possessing end-feet processes, astrocytes contain processes that ensheathe synaptic contacts. The receptors and uptake sites present on astrocytes allow neurotransmitters to communicate with them. These structural and functional characteristics exhibited by astrocytes makes them perfectly suitable to couple local changes in neuronal activity with coordinated adaptations in energy metabolism.

### Glucose metabolism is tightly regulated in all cell types of the brain – neuronal and non-neuronal

Due to enormous degree of cellular heterogeneity in brain, it is quite cumbersome to understand the relative role of each cell type in energy substrate flux. However, usage of primary cultures *in vitro* enriched in neurons, astrocytes or vascular endothelial cells have proved very beneficial in localizing the cellular sites for glucose uptake and its consequent metabolic fate particularly as regards glycolysis and oxidative phosphorylation. As it is well understood that *in situ* cellular preparation does not exhibit all the properties as observed in whole brain tissues many other aspects of brain energy metabolism can be studied using cultures *in vitro*.

Under basal conditions, glucose uptake and its utilization occur in all cell types of the brain with a high specificity. This is due to the presence of unique glucose transporters (GLUTs) on different cell types. Due to low lipid solubility and lack of specific transport carriers in the luminal membrane of the capillary endothelial cell entry of neuroactive compounds such as glutamate, aspartate, and glycine into the blood–brain barrier is limited. Glucose being an obligatory fuel enters through facilitated transport mechanism mediated by specific transporters. Six genes and one pseudogene encoding glucose transporter proteins have been identified so far. These are designated as GLUT1 to GLUT7 (8). In brain, GLUT1, 3, and 5 are preponderantly expressed in cell-specific manner (9). Based on the degree of glycosylation, GLUT1 is expressed in two forms in the brain with molecular weight 55 and 45 kDa (10, 11). The 55 kDa form of GLUT1 is expressed in choroid plexus, ependymal cells, and vascular endothelial cells. The other form with 45 kDa molecular mass is localized on astrocytes (12). Neurons possess GLUT3 transporter on their membrane (13) whereas microglial cells, the resident macrophages of the brain, are found to have GLUT5 form of the transporter (9). So, it is apparent that glucose enters the brain via 55 kDa GLUT1 receptor present on endothelial cells. The uptake by astrocytes is mediated by 45 kDa form of GLUT1 while GLUT3 receptors mediate this process in neurons. Ultimately, microglial cells uptake glucose from the surrounding medium through GLUT5 receptor.

## Glucose Metabolism and Multiple Sclerosis

**Figure 1:**
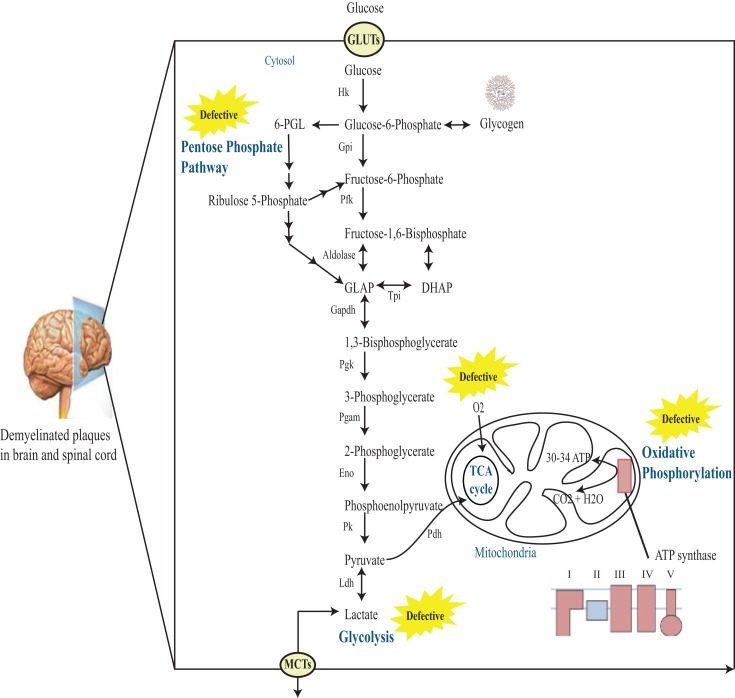
**Schematic representation of glucose metabolism in MS brain**. Glucose enters cells through GLUTs and is phosphorylated by hexokinase to produce glucose 6-phosphate. Glucose 6-phosphate can be processed into three main coordinated metabolic pathways. First, it can be metabolized through glycolysis giving rise to two molecules of pyruvate and producing ATP and NADH. Pyruvate can then enter mitochondria, where it is metabolized through the TCA cycle and oxidative phosphorylation, producing ATP and CO_2_. Alternatively, pyruvate can be reduced to lactate by Ldh. This lactate can be released into the extracellular space through monocarboxylate transporters (MCTs). The complete oxidation of glucose produces 30–34 ATP molecules in the mitochondria. Alternatively, glucose 6-phosphate can be processed through the PPP leading to the production of reducing equivalent in the form of NADPH. Note that the PPP and glycolysis are linked at the level of glyceraldehyde-3-phosphate (GLAP) and fructose 6-phosphate. Finally, glucose 6-phosphate can be converted to glycogen through the process of glycogenesis in astrocytes. Hk, hexokinase; Gpi, glucose-6-phosphate isomerase; Pfk, phosphofructokinase-1; DHAP, dihydroxyacetone phosphate; Tpi, triose phosphate isomerase; GAPDH, glucose-6-phosphate dehydrogenase; Pgk, phosphoglycerate kinase; Pgam, phosphoglycerate mutase; Eno, enolase; Pk, pyruvate kinase; Ldh, lactate dehydrogenase; 6-PGL, 6-phosphoglucono-d-lactone.

Studies show that there is a possible role of impaired energy metabolism in the CNS of MS patients (Figure 1). Initial studies describing the possibility of defective pyruvate metabolism in MS were performed by Jones et al. (14). The group observed elevated blood pyruvate level in both fasting and postprandial times in MS patients with relapse. Similarly, other investigators also reported increased fasting pyruvate level in this disease (15). However, there were conflicting reports demonstrating normal fasting lactate level, or increase in only a small number of patients (16–18). On the other hand, Jeanes and Cumings (16) found an abnormal rise in the blood pyruvate level after glucose intake. These reports hint to a possible abnormality of pyruvate metabolism in MS patients. In addition, increase in levels of Krebs cycle acids like alpha-ketoglutarate in fasting and citrate after glucose intake in MS patients further strengthened the connection of disturbed pyruvate metabolism with MS progression (18). McArdle et al. (19) found elevated levels of pyruvate and α-ketoglutarate in MS. Increased activity of metabolic enzymes including enolase, pyruvate kinase, lactate dehydrogenase (Ldh), and aldolase in the CSF of patients with disseminated sclerosis make them a sensitive indicator of active demyelination (20).

B cells and antibodies reactive with triose phosphate isomerase (TPI) and GAPDH are produced intrathecally in CSF and lesions of MS (21). Both TPI and GAPDH are essential metabolic enzymes involved in ATP production. Another investigation by the same group showed that these antibodies bind with TPI and GAPDH and inhibit the glycolytic activity of GAPDH but not TPI in MS patients (22). This inhibitory effect of antibodies on GAPDH was not visualized when anti-GAPDH IgG was exhausted from the CSF demonstrating the role of anti-GAPDH antibodies in impeding the GAPDH enzyme activity in brain leading to neuronal apoptosis and cytotoxicity. In systemic lupus, an autoimmune rheumatoid disorder, autoantibodies were found reactive to GAPDH (23). Accumulating evidence indicates that inhibition of GAPDH activity in glycolytic pathway is associated with neuronal apoptosis. It has been purported that GAPDH enzyme activity suppresses when it reacts with other proteins in the CNS (24). Single chain variable fragment antibodies (scFv-abs) obtained from clonally expanded B cells binds specifically with GAPDH and TPI in active MS lesions (25). In addition, interaction of GAPDH with ß-amyloid protein and Huntingtin protein well demonstrates the role of GAPDH in neurodegenerative disorders (26).

A recent study demonstrated that a ligand-activated transcription factor known as peroxisome proliferator-activated receptor gamma (PPARγ) playing a pivotal role in regulation of glucose and lipid metabolism is markedly increased in CSF of MS patients (27). Furthermore, elevated expression of PPARγ has been reported within the spinal cord of EAE mice (28) and in an *in vitro* model of antigen induced demyelination (29). These findings may contribute to our understanding about the role of PPARγ in the pathogenesis of MS. William et al. studied the role of CNS energy metabolism in MS disease progression. The group measured the levels of lactate, sorbitol, and fructose, all metabolites of extra-mitochondrial glucose metabolism, in the CSF of relapsing remitting and secondary progressive MS patients (30). Sorbitol and fructose are the metabolites of polyol pathway that run parallel to glycolysis and lactate is the metabolite of anaerobic pathway. The finding demonstrated elevated levels of all three metabolites in the CSF of SPMS patients and to a lesser extent to RRMS patients (Figure S1 in Supplementary Material). These alterations in energy metabolism may contribute to mitochondrial dysfunction and neuroaxonal degeneration underlying MS progression. Taken together, the finding supports a link between increased activity of extra-mitochondrial pathways of glucose metabolism and MS disease progression. Other studies found that the activity of enolase, pyruvate kinase, Ldh, and aldolase, all metabolic enzymes was increased in disseminated sclerosis (20).

A recent investigation demonstrated differences in the gene expression levels of various NADPH subunits between initial MS lesions and control white matter brain. The group performed whole genome profiling of MS brain tissue and observed significant up regulation of nicotinamide dinucleotide phosphate oxidase I and nicotinamide dinucleotide phosphate oxidase organizer I in active MS lesions (31). NADPH oxidase is a multi-subunit enzyme complex that is activated under pathological conditions in microglia and catalyzes the production of superoxide from O_2_. Other studies have identified defects in mitochondrial electron transport gene expression and function in postmortem MS cortex (32, 33). These studies have found transcriptional changes in important mitochondrial genes; however, translational or post-translational changes in several other proteins may also influence mitochondrial function and energy production.

## Mitochondrial Defects in MS

Mitochondrial dysfunction is implicated in various pathological conditions like diabetes, anxiety disorders, neurodegenerative diseases such as Alzheimer’s, Huntington’s, and Parkinson’s disease, and cancer and fatigue (see for instance Table 1). It is only recently that mitochondrial aberrations have been studied in MS. Unlike nuclear DNA, mitochondrial DNA (mtDNA) is not surrounded by histones, proteins that shield nuclear DNA from free radicals. Therefore it is quite prone to damage. The number of mitochondria in a cell is determined by its energy requirement. For instance, there may be up to 200–2000 mitochondria present in a single somatic cell whereas the number is fixed to 16 in spermatozoa germ cells and 100,000 in oocytes. Metabolically active cells like skeletal muscle, cardiac muscle, and brain contain largest number of mitochondria. It has been seen that the number of mitochondria and their activity is increased in MS plaques (34). In demyelinating diseases particularly MS, it is likely that cells need ample energy to survive. Hence, metabolic activity of biomolecules in mitochondria increases with concomitant impairment of Krebs cycle and/or neuronal oxidative phosphorylation within the CNS. Another study revealed a reduction in ATP synthase expression in MS lesions (35). Mitochondrial proteins are expressed in greater amounts in both active and inactive lesions. Activity of complex IV found on mitochondrial membrane is increased dramatically in MS lesions (30). Lu et al. (36) revealed defects in complex I component of ETC in white matter lesions. Furthermore, reduced functional activity of complex I and complex III and a decrease in gene expression of complex I, complex III, complex IV, and ATP synthase has been observed in non-lesional motor cortex (33).

**Table 1:** **Comparison of disturbed glucose metabolism in MS and other neurodegenerative disorders**.

Glucose metabolism	Multiple sclerosis	Other neurodegenerative disorders
Glycolysis	Elevated blood **pyruvate level** was observed in both fasting and postprandial times in MS patients with relapse (14)	Impaired **GAPDH** function was observed in subcellular fractions of fibroblasts from Alzheimer and Huntington patients (37) **GAPDH** was found to be overexpressed in the neocortex and caudate putamen neurons in a transgenic model of Huntington’s disease (38) The activity of **GAPDH, hexokinase and pyruvate kinase** was increased in Alzheimer’s disease (AD) (39)
	**Pyruvate levels** were found to be increased in MS (19)	
	The activity of metabolic enzymes including **enolase and pyruvate kinase** was found to be increased in the CSF of MS patients (20)	
	Antibodies reactive with **triose phosphate isomerase (TPI) and GAPDH**, bind with them and inhibit the glycolytic activity of GAPDH in MS patients (22)	
	The levels of **enolase, pyruvate kinase, Ldh, and aldolase**, all metabolic enzymes were increased in MS (20)	
TCA cycle	Krebs cycle proteins like **α-ketoglutarate levels** in fasting and **citrate levels** after glucose intake were found to be increased in MS patients (18)	Mitochondrial **aconitase, succinyl-CoA synthetase β, fumarase** and **malate dehydrogenase** showed decreased gene expression in hippocampal samples from autopsy AD brains in two independent studies (40, 41) Mitochondrial **oxoglutarate dehydrogenase**, showed increased and decreased gene expression in moderate and severe AD patients (40)
	**α-Ketoglutarate levels** were found to be increased in MS (19)	
	Krebs cycle enzyme **aconitase** activity was found to be higher in MS patients without fatigue (42)	
Oxidative phosphorylation	A reduction in the expression of **ATP synthase** gene was observed in MS lesions (35)	Decreased mRNA expression levels of ETC proteins, specifically **FAD synthetase, riboflavin kinase (RFK), cytochrome C1 (CYC1)**, and **succinate dehydrogenase** complex subunit B were reported in amyotrophic lateral sclerosis (43) Decreased mRNA levels of the mitochondrial-encoded **cytochrome oxidase (COX) subunits I, II, and III** were observed in brains of AD patients (41, 44, 45) Reduced expression of nuclear encoded subunits of mitochondrial enzymes of oxidative phosphorylation including **subunit IV of COX** and the **beta-subunit of the F0F1-ATP synthase** was also observed in vulnerable areas of AD brains (41, 44)
	Activity of mitochondrial ETC **complex IV** was increased dramatically in MS lesions (30)	
	Defects in **complex I** component of mitochondrial ETC were observed in white matter lesions (36)	
	Furthermore, reduced functional activity of **complex I and complex III** and a decrease in gene expression of **complex I, complex III, complex IV, and ATP synthase** has been observed in non-lesional motor cortex (33). In contrast, enzyme activities of **complex I, II, III, IV, and V** were found to be higher in MS patients compared to controls. **Complex II** activity increased significantly in MS group between patients with and without fatigue (42)	
	A significant reduction in the gene expression of **cytochrome c oxidase 5B subunit (COX5B)** was observed in MS patients (46)	
	Analysis of a number of respiratory chain proteins reveals functionally important defects of mitochondrial proteins [**cytochrome c oxidase (COX)** and its catalytic component, **COX-1**] in complex III in MS (47)	
	Different expression of mitochondrial proteins namely **cytochrome c oxidase subunit 5b (COX5b), hemoglobin β, creatine kinase, and myelin basic protein (MBP)** was found in the brain of MS (48)	

A recent finding demonstrated changes in mitochondrial complex enzyme activities and cytochrome c expression in platelets of MS patients. Krebs cycle enzyme aconitase activity was higher in patients without fatigue and all respiratory complex enzyme activities (complex I, II, III, IV, and V) were higher in MS patients compared to controls. Complex II activity increased significantly in MS group between patients with and without fatigue (42). Interestingly, a significant reduction in the gene expression of cytochrome c oxidase 5B subunit (COX5B) was observed in MS patients (46). This data suggest that there is a down regulation of genes associated with mitochondrial ETC. Analysis of a number of respiratory chain proteins reveals functionally important defects of mitochondrial proteins [cytochrome c oxidase (COX) and its catalytic component, COX-1] in complex III in MS (47). Different expressions of mitochondrial proteins namely cytochrome c oxidase subunit 5b (COX5b), hemoglobin ß, creatine kinase, and myelin basic protein (MBP) were found in the brain of MS (48). Taken together, these studies show mitochondrial abnormalities that may cause functional disturbance in the surviving demyelinated axons in MS and may result in neurological dysfunction.

## Glucose Metabolism and Neurodegenerative Disorders

A body of evidence indicates a link between disturbed metabolic function and the progression of neurodegenerative diseases like AD, Huntington’s disease and Parkinson’s disease. A report documented that GAPDH glycolytic function is impaired in subcellular fractions of fibroblasts from Alzheimer and Huntington patients whereas the gene expression remained unchanged (37). This might have occurred due to post-translational modification of the GAPDH protein. In a transgenic model of Huntington’s disease, GAPDH is seen to be overexpressed in specific neuronal populations of several brain regions, such as the neocortex and caudate putamen neurons. This study also revealed translocation of GAPDH into the nucleus and the subsequent cell loss in the neocortex and caudate putamen region of the brain (38). Similar finding was observed by Bae et al. (49), who demonstrated that GAPDH facilitates nuclear translocation of mutant Huntingtin protein (mHtt) and causes neurotoxicity. In Aβ (amyloid beta) resistant cells of Alzheimer’s brain, glycolytic pathway was upregulated and hexose monophosphate shunt (HMS) was activated. The activity of GAPDH, hexokinase, and pyruvate kinase was increased in both glycolysis and HMS (39). In amyotrophic lateral sclerosis (ALS), abnormalities in ETC have been reported. The study found decreased mRNA expression levels of ETC proteins, specifically FAD synthetase, riboflavin kinase (RFK), cytochrome C1 (CYC1), and succinate dehydrogenase complex subunit B (SDHB) (43).

## Conclusion

Despite extensive research being carried out for a decade the underlying cause of MS still remains elusive. Perturbed glucose metabolism is implicated in neurodegenerative disorders like Alzheimer’s, Parkinson’s, and Huntington’s. However, little is known about its role in MS pathology. The observations reviewed in this article, especially those referred with mitochondrial aberrations and impaired glucose metabolism in MS, pointed to a relationship between glucose metabolism and MS disease pathogenesis. Although traditionally considered as an autoimmune, inflammatory, and demyelinating disease of the CNS, the scenario of MS pathogenesis associated with metabolic abnormalities is speculated.

These observations open new perspectives for the understanding of metabolic dynamics in MS yet many puzzling aspects and critical questions need to be addressed. For instance, how does defect in metabolic pathway contributes to demyelination? Does metabolic pathway alter in other cell types in MS? The changes in a cell type, may affect directly, through some unknown factor, or indirectly, through global changes in metabolic intermediates levels in CSF, to other cell types? Is disturbance in metabolic pathway a mere cause or a consequence of MS? Could those genes be used as a pharmacological target to alleviate MS pathology? Much more research is required to fully unravel the disease mechanism, and a proper understanding of the disease could eventually lead to new treatments.

## Conflict of Interest Statement

The authors declare that the research was conducted in the absence of any commercial or financial relationships that could be construed as a potential conflict of interest.

## Supplementary Material

The Supplementary Material for this article can be found online at http://www.frontiersin.org/Journal/10.3389/fneur.2014.00250/abstract

## Abbreviations

CNS, central nervous system; ETC, electron transport chain; FAD, flavin adenine dinucleotide; MS, multiple sclerosis; NAD^+^, nicotinamide adenine dinucleotide; RRMS, relapsing-remitting multiple sclerosis; SPMS, secondary progressive multiple sclerosis; TCA cycle, tricarboxylic acid cycle.
