# Supplementary figures and images for: Perturbed Glucose Metabolism: Insights into Multiple Sclerosis Pathogenesis
Source: Front Neurol. 2014 Dec 1;5:250. doi: 10.3389/fneur.2014.00250 (PMC4249254; doi:10.3389/fneur.2014.00250)

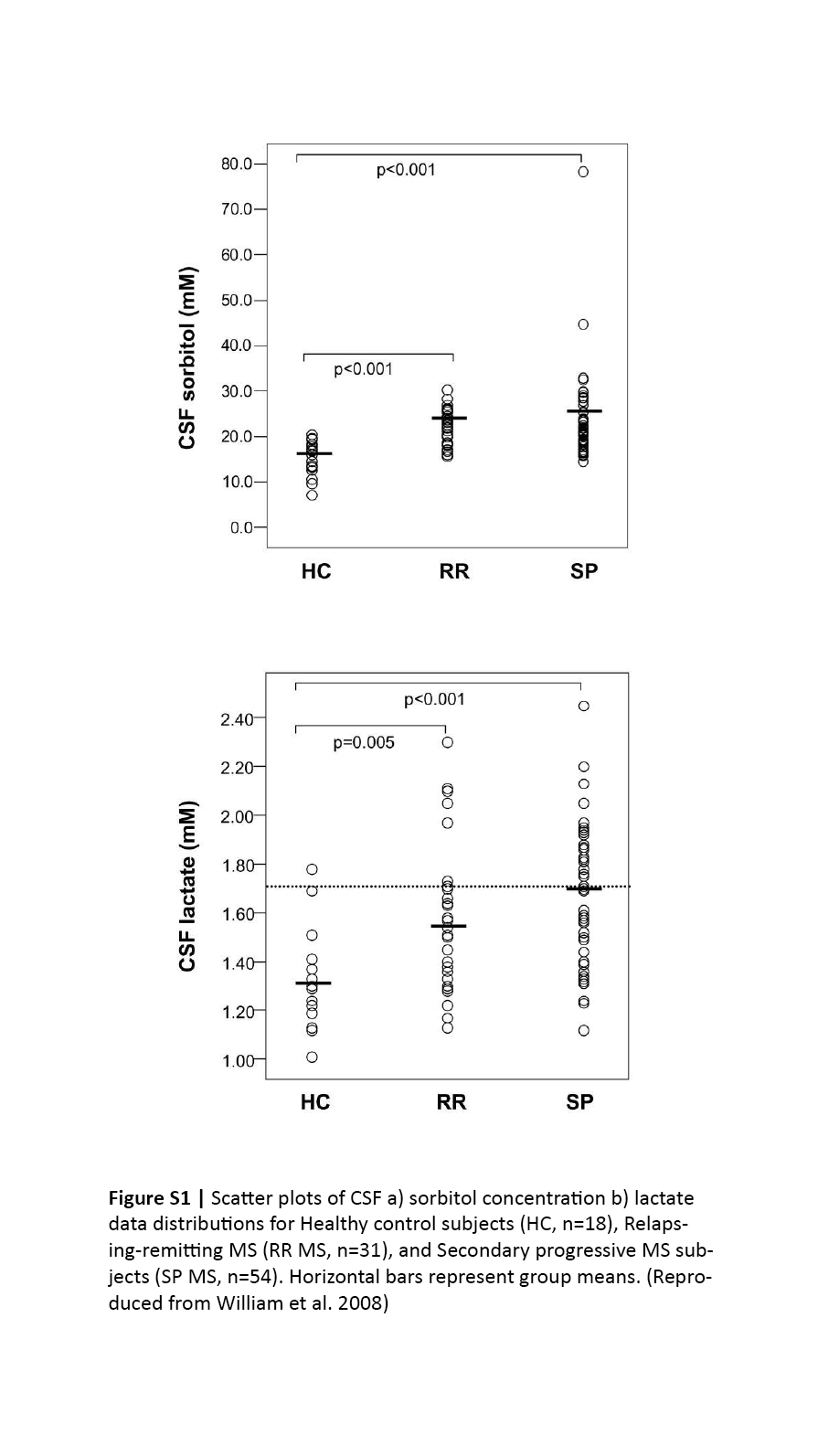

Supplement: Supplementary file 1 [file Image1.TIF]
